# Breast Amyloidosis: A Case Report and Literature Review

**DOI:** 10.5334/jbsr.2988

**Published:** 2022-12-19

**Authors:** Anne-Sofie De Crem, Koen Van de Vijver, Pieter De Visschere, Rudy Van den Broecke, Menekse Göker

**Affiliations:** 1 UGent, BE; 2 Ghent University Hospital, BE

**Keywords:** Localized amyloidosis, breast, microcalcifications, mammography, lupus erythematosus

## Abstract

Amyloidosis is an uncommon disorder characterized by extracellular accumulation of misfolded proteins in tissues. We report a unique case of localized breast amyloidosis in an asymptomatic 56-year-old woman with systemic lupus erythematosus, presented as suspicious microcalcifications without mass on mammography. Vacuum biopsy confirmed amyloidosis, producing the typical apple-green birefringence under polarized light after staining with Congo-red. Further workup and follow-up to exclude development into systemic amyloidosis or hematologic malignancy is recommended. If negative, the prognosis is very good, thus no further treatment is needed. A brief review of the literature revealed more about the typical findings and recommended management.

## Introduction

Amyloidosis of the breast is a very rare condition that was reported for the first time in 1973 by Fernandez et al. and ever since only in isolated case reports and few case series [1]. Amyloidosis is an uncommon disorder characterized by extracellular deposition of amyloids. Amyloids are proteins that were folded incorrectly into an abnormal fibrillar, β-pleated sheet [2], due to their precursor proteins that either have an abnormal structure or are enormously increased in the serum. Their abnormal folding makes it difficult to be broken down by proteases, so they accumulate. Breast amyloidosis can be part of systemic amyloidosis or may be limited to the breast, almost equally divided [3].

Systemic amyloidosis is characterized by deposits, in multiple organs, which disrupt the organ structure, ultimately leading to organ dysfunction with associated symptoms, end organ failure and death. The most common systemic forms are either accumulation of immunoglobulin light chains (AL or primary), such as multiple myeloma or accumulation of serum protein A (AA or secondary) from the acute phase response associated with chronic inflammation (in RA, IBD, TBC or cancers) [3,4]. Breast involvement in case of systemic amyloidosis occurs most frequently in advanced disease, mostly defined as the AL (kappa) type [3,5].

In localized amyloidosis (LA), only one organ is affected. It is a rare entity only described in 13% of all amyloid cases evaluated by Röcken et al. and predominantly identified in the larynx, airway, bladder, colon and skin [5,6]. Usually the AL type (light chains from mucosal lymphoid tissue, mostly kappa) is reported [3]. Known examples of the localized form are Alzheimer disease (Aß peptides plaques interfering with signaling), diabetes mellitus type 2 (Amylin deposits) or familial amyloid CMP (transthyretin mutant causing ATTR amyloidosis). Localized amyloidosis of the breast is a very rare variant, only seen in 0.5% of patients diagnosed with amyloidosis over 18 years [5].

Differentiation between systemic and localized amyloidosis is essential to determine further management (i.e., chemotherapy against the underlying disease in systemic forms [5]). Here, we report an interesting case of bilateral localized breast amyloidosis, followed by a brief literature review.

## Case History

**Figure 1:**
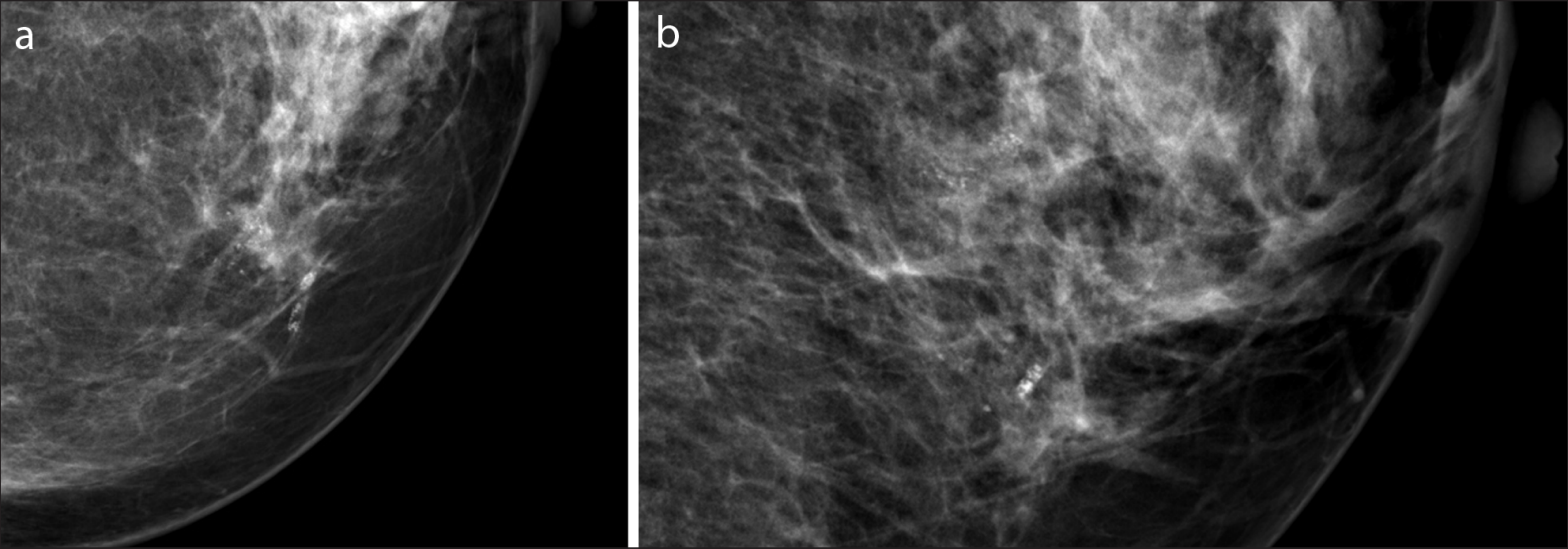
Mammography of the left breast in 2016 shows a group of new micro- and macrocalcifications (irregular pleiomorphic, tubular and linear) of 2 cm at 9h (**a:** medial part on craniocaudal incidence, **b:** caudal part on medial-oblique incidence). No associated architectural distortion or mass.

A 56-year-old asymptomatic Caucasian woman visited the outpatient clinic for a routine control in July 2016, combined with a screening mammography. She was known to have systemic lupus erythematosus for more than thirty years with several systemic complications such as lupus nephropathy, meningitis, vasculitis and secondary Raynaud syndrome. The gynaecological examination showed no abnormalities. However, the mammography in the left breast showed new calcifications in comparison to the screening mammography of 2013 (see Figure 1). These lesions where suspicious for a ductal carcinoma in situ (DCIS), thus the patient underwent a vacuum assisted stereotactic breast biopsy. On ultrasonography no abnormalities were observed.

**Figure 2:**
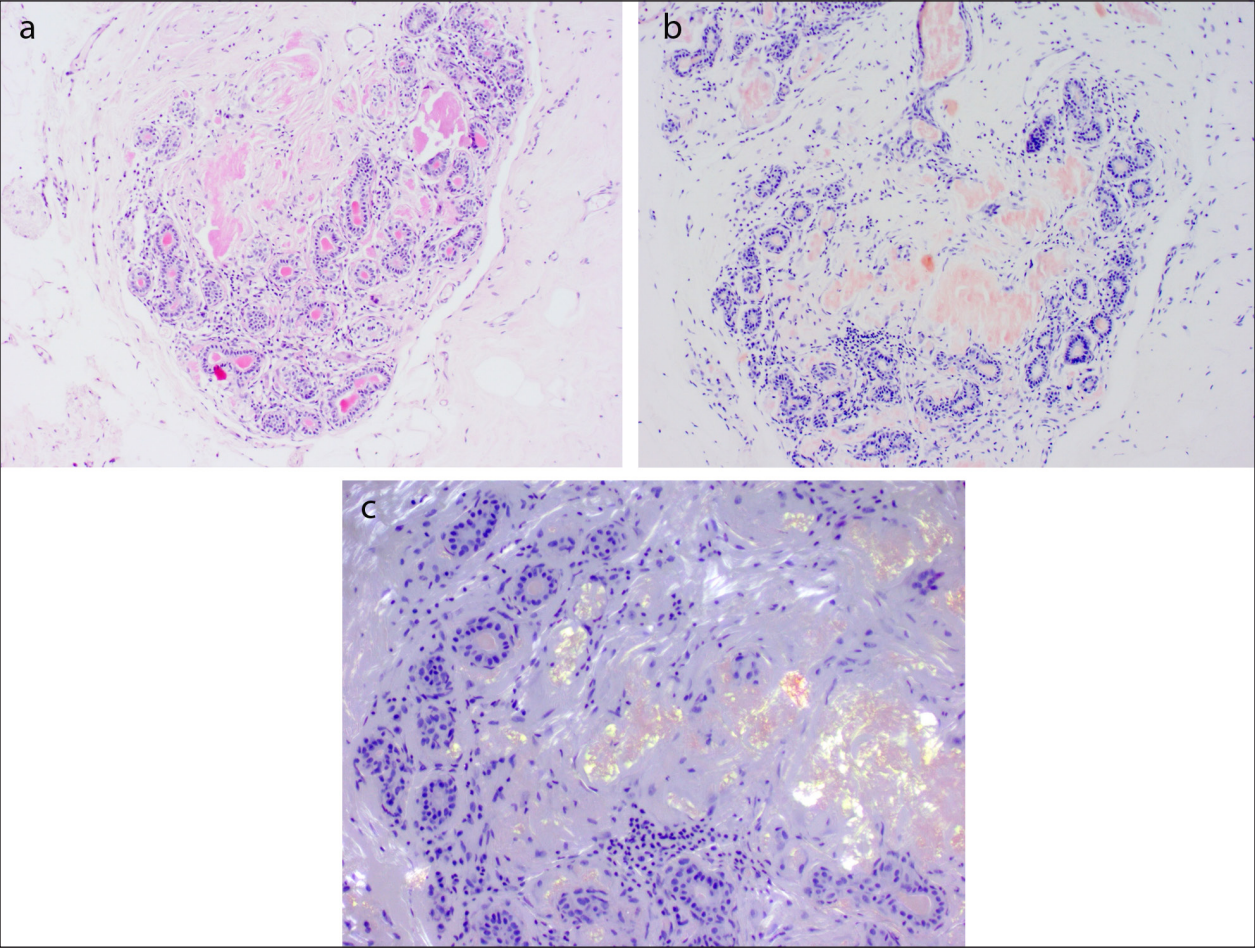
APD amyloid deposition. **a.** Hematoxylin and eosine staining, amyloid is bright pink. **b.** Congo red, amyloid is red. **c.** Congo red under polarized light, amyloid is apple-green.

The biopsy showed no evidence of malignancy. Staining with Congo-red, however, showed the typical apple-green birefringence under polarized light that indicates the presence of several amyloid depositions (see Figure 2). Further staining for kappa and lambda (AL type) was negative, whereas Amyloid A and transthyretin were positive suggesting the diagnosis of AA type amyloidosis. Subsequently, a workup to rule out systemic amyloidosis using protein electrophoresis was done and found negative. Other laboratory tests were only positive for anti-dsDNA (68.21 IU/L) and ANA, consistent with the diagnosis of SLE. No malignancy or concomitant hematologic disorder was found.

A harpoon guided excision of these microcalcifications in the left breast, was performed and confirmed amyloidosis without breast cancer.

**Figure 3:**
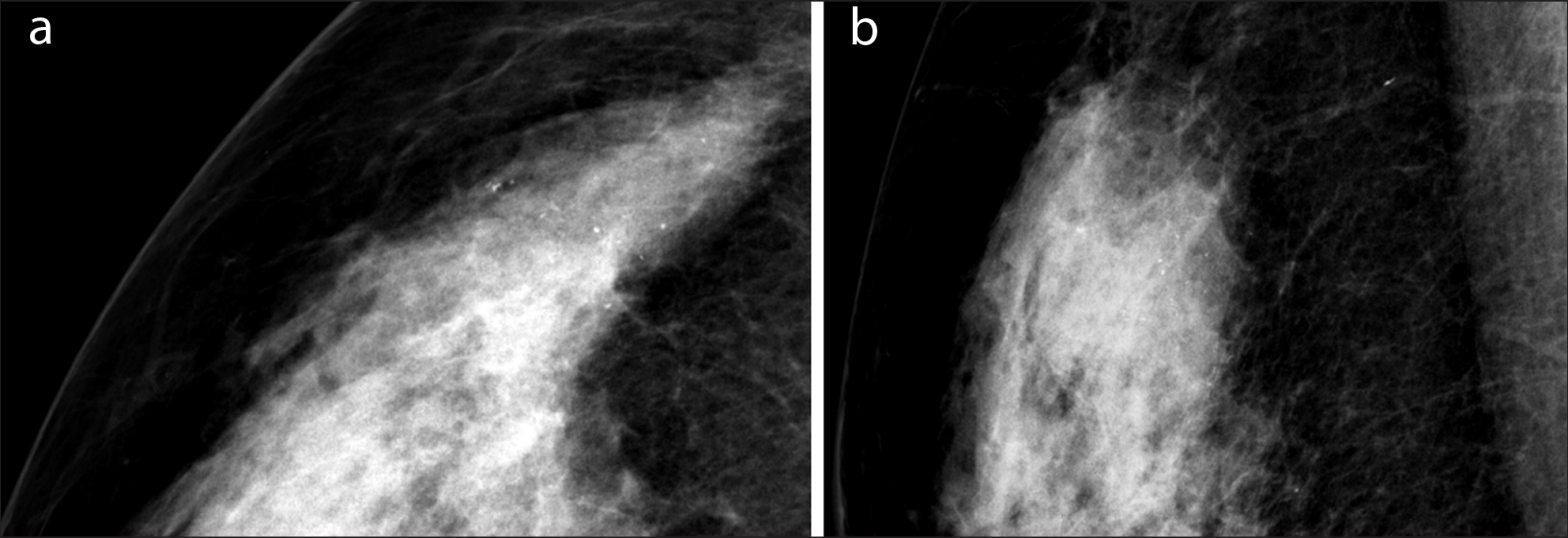
Mammography of the right breast in 2018 shows new micro- and macrocalcifications (fine regular and punctiform) of 2 cm at 11h (**a:** lateral part on craniocaudal incidence, **b:** cranial part on medial-oblique incidence). No architectural distortion or mass.

In conclusion, there was no further evidence for a DCIS or other malignancy neither for a systemic type of amyloidosis, so the diagnosis of unilateral localized amyloidosis of the breast was withheld. Considering its benign nature, no further treatment was given. This was the first manifestation of amyloidosis in this patient, who did however have a long history of an inflammatory condition caused by SLE. Follow-up of 6 years with yearly serum electrophoresis showed no clinical or radiological manifestations that suggests a progression to generalized or extramammary amyloidosis. Nevertheless, the screening mammography of 2018 showed new calcifications in the right breast and amyloidosis (Amyloid A) was confirmed on vacuum biopsy again (see Figure 3).

## Comment

This case is unique due to the rarity of bilateral localized amyloidosis in the breast, the early age of diagnosis, the concomitant systemic lupus erythematosus disease and AA type amyloidosis but mostly the presentation on mammography showing only microcalcifications. Literature shows that amyloidosis of the breast is very rare (0.5% of amyloid cases) and it manifests more frequently in postmenopausal women with mean age of 63 years [5,6,7]. Usually, women are asymptomatic (painless, not palpable mass) and it is therefore only found on routine screening [6]. Other clinical signs can be a mass lesion, peau d’orange or a general tenderness [3,5]. It is mostly seen unilateral without preference for the right or left breast [3,5].

The typical presentation of breast amyloidosis on mammography consists of solitary or multiple breast masses, with or without calcifications [5,7]. Only a few case reports, including this one, exist reporting microcalcifications as sole presentation without any mass [8,9]. These calcifications derive from the amyloid protein that has a calcium affinity which accumulates in perivascular, periductal and intralobular areas in the breast tissue [5,6]. Calcium accumulations in these areas are seen on mammography images as thin linear, branching, bar-shaped, pleomorphic or cluster-forming micro- and macrocalcifications [8]. As no clinical or radiological signs are specific for amyloidosis and a malignancy needs to be excluded, a diagnostic biopsy or excision is inevitable.

The diagnosis of amyloidosis is thus confirmed with the typical Congo-red staining of the biopsy showing apple-green birefringence under polarized light. The incidence of amyloidosis could probably be much higher than suspected today if breast biopsies, taken to exclude malignancy, would systematically be stained with Congo-red.

Unlike our case, associated malignancies were reported like DCIS (most frequently) but also ductal or lobular carcinoma [3,5,6]. The reported association for invasive breast cancers differs from 0% by Said et al to 14% by Charlot et al. and Röcken et al. (1 in 7 patients and 6 in 43 patients, respectively) [3,5,6]. Almost half (47%) of the patients with breast amyloidosis have systemic amyloidosis, and more than half (55%) have concurrent hematologic disorders (mostly MALT next to non-Hodgkin lymphomas) [3]. Therefore, work up to rule out systemic diseases is recommended. This consists of further staining of the biopsy with the most common systemic precursor proteins, such as Amyloid A for AA type or kappa and lambda for AL type. The dominance of the AL type (in 96%) suggests that local amyloidosis originates from local plasma cells secreting immunoglobulins [3]. Nevertheless, breast amyloidosis may also be related to chronic inflammatory diseases such as rheumatoid arthritis or Sjögren’s syndrome, in which the AA type is expected but not always confirmed [5]. This hypothesis may suspect an AA type in this woman with SLE, although no case of this kind was yet reported. Besides staining, further clinical investigations should be performed such as protein electrophoresis or immunofixation electrophoresis on serum or urine [6]. In most cases, including this one, these investigations were inconclusive [3,10]. Furthermore, taking biopsies of specific predominant sites of amyloidosis (lymph nodes, GI tract, abdominal adipose tissue) have been suggested in some cases [5]. According to Charlot et al., this workup should be completed with an electrocardiogram, echocardiogram, chest radiography and abdominal fat pad aspirate for Congo-red staining [6]. Finally, a free light chain assay and bone marrow biopsy might also be done.

It is a benign disease with a good prognosis, thus no further treatment is necessary [3]. The prognosis for localized breast amyloidosis is much better than the systemic type, considering most patients die of complications of lymphoma or leukemia (41% died after less than 3 years of follow-up) [3]. In literature, unilateral mammography is recommended six months after surgical excision, followed by the annual routine if no pathology was seen except postoperative changes and scar tissue [7]. Only rarely systemic amyloidosis or haematological disorders will develop in localized breast amyloidosis thus no further investigations were recommended. This case report confirms no need for yearly serum electrophoresis seeing this stayed normal over six years of follow-up but the need for screening mammography which detected more amyloidosis in the right breast after two years.

## Conclusion

We report a case of metachronous bilateral localized amyloidosis in the breast, presenting as microcalcifications only on mammography. Vacuum biopsy with histopathological examination and Congo-red staining under polarized light is needed for diagnosis. Extensive surgery can be avoided in most cases given its benign nature, but further work-up should include a search for systemic amyloidosis or concurrent local or hematological disorders.

## Limitations

Only limited cases of breast amyloidosis were reported. This makes it rather difficult to improve the knowledge of its clinical, radiological and histopathological presentation and therefore optimize the further workup to distinguish the type of amyloidosis. Consequently, there is a need to collect and report more cases of breast amyloidosis, preferably as a case series.
